# Immune landscape and immunotherapy for penile cancer

**DOI:** 10.3389/fimmu.2022.1055235

**Published:** 2022-11-29

**Authors:** Yaxiong Tang, Xu Hu, Kan Wu, Xiang Li

**Affiliations:** Department of Urology, Institute of Urology, West China Hospital, Sichuan University, Chengdu, China

**Keywords:** penile cancer, tumor immune microenvironment (TIME), immunotherapy, human papillomavirus (HPV), immune checkpoint inhibitor (ICI)

## Abstract

Penile cancer is a rare malignancy and usually refers to penile squamous cell carcinoma (PSCC), which accounts for more than 95% of all penile malignancies. Although organ-sparing surgery is an effective treatment for early-stage PSCC, surgical intervention alone is often not curative for advanced PSCC with metastases to the inguinal and/or pelvic lymph nodes; thus, systemic therapy is required (usually platinum-based chemotherapy and surgery combined). However, chemotherapy for PSCC has proven to be of limited efficacy and is often accompanied by high toxicity, and patients with advanced PSCC usually have poor prognosis. The limited treatment options and poor prognosis indicate the unmet need for advanced PSCC. Immune-based therapies have been approved for a variety of genitourinary and squamous cell carcinomas but are rarely reported in PSCC. To date, several studies have reported high expression of PDL1 in PSCC, supporting the potential application of immune checkpoint inhibitors in PSCC. In addition, human papillomavirus (HPV) infection is highly prevalent in PSCC and plays a key role in the carcinogenesis of HPV-positive PSCC, suggesting that therapeutic HPV vaccine may also be a potential treatment modality. Moreover, adoptive T cell therapy (ATC) has also shown efficacy in treating advanced penile cancer in some early clinical trials. The development of new therapeutics relies on understanding the underlying biological mechanisms and processes of tumor initiation, progression and metastasis. Therefore, based on the interest, we reviewed the tumor immune microenvironment and the emerging immunotherapy for penile cancer.

## Introduction

Penile cancer is a rare malignancy with approximately 26,000 new cases worldwide per year; despite the low overall incidence of approximately 1/100,000 in developed countries, the incidence is much higher in developing countries (1–3). Penile cancer usually refers to penile squamous cell carcinoma (PSCC), which comprises more than 95% of all penile malignancies; other penile malignancies, such as melanocytic lesions, mesenchymal tumors, lymphomas, and metastases, are less common (4, 5). Based on the current knowledge, phimosis, chronic inflammation of the penis, smoking, lower socioeconomic status, ultraviolet exposure, and human papillomavirus (HPV) infection are regarded as risk factors for penile cancer (6–11). In addition, approximately 30% of penile intraepithelial neoplasia (PeIN), which is an unfavorable histopathological feature associated with penile cancer, will progress to invasive penile cancer if untreated (12).

With non-inferior 5-year survival compared with radical surgery, organ-sparing surgery alone is recommended as the primary curative treatment for PeIN and localized invasive penile cancer by the guidelines of the European Association of Urology (EAU) and National Comprehensive Cancer Network (NCCN) (5, 13). However, despite not affecting overall survival (OS), the probability of recurrence after organ-sparing surgery is high, and penectomy will then be inevitable for some patients. A retrospective study of 203 PSCC reported that 18% of patients had local recurrence after organ-sparing surgery, of whom approximately 17% required penectomy (14). As a result of penectomy, patients’ sexual life and overall well-being will be significantly affected (15). The survival outcomes of patients diagnosed with advanced PSCC are affected by multiple factors (such as subtypes of pathology, perineural and lymphovascular involvement, and extracapsular spread of lymph node metastasis), and surgery alone is usually noncurative in this setting (16, 17). With curative intent, the NCCN guideline recommends 4 cycles of neoadjuvant chemotherapy (NAC) with a combination of paclitaxel, ifosfamide, and cisplatin (TIP) for patients with inguinal lymph node(s) larger than 4 cm or patients who are at the N2/N3 stage, while adjuvant chemotherapy (AC) is recommended for patients with high-risk features (pelvic lymph node metastases, extranodal extension, bilateral inguinal lymph nodes involved, 4 cm tumor in lymph nodes) (13). Unfortunately, chemotherapy was proven to have limited benefits for PSCC patients, and the prognosis for advanced PSCC is unsatisfactory with current treatment options. In a phase 2 trial that included 30 patients diagnosed with advanced N2/N3 stage PSCC without distant metastases, 4 cycles of NAC of TIP resulted in a 50% objective response rate, 22 (73.3%) patients underwent surgery after NAC, and the median progression months and median survival months were only 8.1 months (95% confidence interval [CI], 5.4 to 50 months) and 17.1 months (95% CI, 10.3 to 60 months), respectively (18). Other studies have reported several additional moderately efficacious and often highly toxic chemotherapy regimens for locally advanced or metastatic PSCC (19–22). Moreover, the treatment options available after chemotherapy failure are few and often have poor efficacy. Based on a retrospective study, patients with advanced PSCC had a poor response to salvage therapy after first-line chemotherapy failure, with a median OS of less than six months (14). The limited treatment options and poor prognosis indicate an unmet need for systemic therapy for penile cancer.

Immune-based therapy has been approved for the treatment of numerous genitourinary carcinomas (23–30). Pembrolizumab, which is a kind of immune checkpoint inhibitor (ICI), is recommended by the NCCN guidelines as the second-line treatment for unresectable or metastatic PSCC with high tumor mutational burden (TMB-H) or deficient mismatch repair (dMMR). However, the few and mainly case reports and basket trial data on the effect of pembrolizumab on clinical outcomes limited its widespread use in lethal advanced PSCC (31–34). Just as higher expression of PDL1 correlates with improved response to ICI in other tumors (35), the high PDL1 expression rate in PSCC tissue suggests that ICI may be a potentially effective treatment for PSCC (36). In addition, the distinct molecular mechanisms and prognosis between HPV-positive and HPV-negative PSCC make HPV-related therapies, such as therapeutic HPV vaccines, a potential focus for penile cancer treatment (37, 38). Moreover, adoptive T cells therapy (ATC) has also shown efficacy in treating advanced penile cancer in some early clinical trials, also emerging as a potential treatment for penile cancer (36).

The development of new therapeutics relies on understanding the underlying biological mechanisms and processes of tumor initiation, progression and metastasis. A recently published review provided a systematic review of immune-based therapies in penile cancer. However, basic concepts (such as tumor immunity, tumor mutation burden, microsatellite instability, etc.), the history of urogenital tumor immunotherapy, the carcinogenesis of penile cancer, and the association between HPV infection and penile cancer seem to be inadequate in this review. Therefore, based on interest, we provided an overview of immune landscape and immunotherapy for penile cancer, hoping to complement previous studies to better and more fully understand the prospects of immunotherapy for penile cancer.

## Tumor immune microenvironment

### Carcinogenesis of penile cancer

HPV, especially HPV16 infection, is common in penile cancer and contributes to the carcinogenesis of penile cancer. In a systematic review and meta-analysis that included 71 studies, HPV DNA was detected in 50.8% (95% CI, 44.8%–56.7%) of invasive penile cancers and 79.8% (95% CI, 69.3%–88.6%) of PeIN, with basaloid squamous cell carcinoma and warty carcinoma becoming the histological subtypes with the top 2 highest HPV infection rates, reaching 84.0% (95% CI, 71.0%-93.6%) and 75.7% (95% CI, 70.1%–81.0%), respectively (39). In addition, the prevalence of HPV infection in penile cancer samples varied between regions, ranging from 40% in a Spanish cohort of 82 PSCC cases (40) to 90.2% in a South African cohort of 66 PSCC cases (41). Due to the different mechanisms of carcinogenesis between HPV-positive and HPV-negative penile cancer (37, 42, 43), the World Health Organization (WHO) fourth edition of the genitourinary cancer classification divided penile cancer into HPV-related and non-HPV-related penile cancer based on the presence or absence of HPV infection; the former mainly includes basaloid squamous cell carcinoma and warty carcinoma, while the latter mainly includes PSCC of the usual type (44, 45). Notably, PSCC of the usual type, the most common histological subtype of penile cancer, although classified as non-HPV-related penile cancer, approximately one-third of them are associated with HPV infection, usually HPV16 (44). Interestingly, compared with HPV-negative PSCC, several observational studies and meta-analyses found that HPV-positive PSCC has a better survival, which may be due to the different oncogenic mechanisms and tumor immune microenvironment (TIME) between HPV-positive and HPV-negative PSCC (37, 46–49).

**Figure 1:**
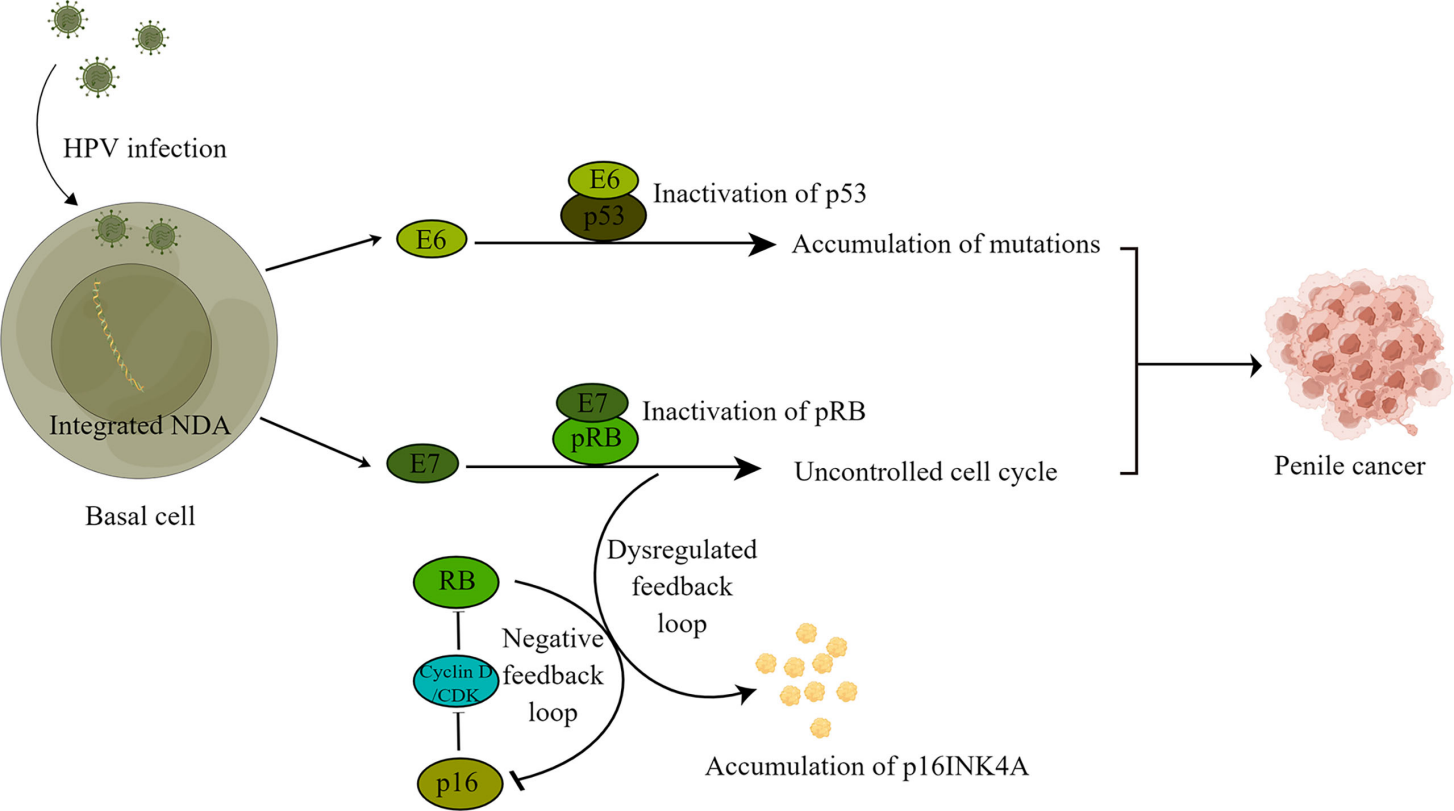
Carcinogenesis mechanism of HPV-mediated penile cancer. Human papillomavirus infects basal cells and integrates its own DNA into host DNA, resulting in high expression of viral oncoproteins E6 and E7. High expression of E6 and E7 leads to the inactivation of retinoblastoma protein (pRB) and p53, resulting in the accumulation of mutations and an uncontrolled cell cycle, ultimately leading to tumorigenesis. In addition, disruption of the negative feedback between p16INK4A and pRB due to the inactivation of pRB leads to high-level expression of p16INK4KA in penile cancer.

The oncogenic mechanism of HPV-positive PSCC has been well described (**Figure 1**). Through microabrasions and specific receptors such as heparan sulfate proteoglycan and α6 integrin, HPV can infect the basal cells of the epithelial mucosa and then integrate HPV DNA into the host genome, thus resulting in high levels of viral oncoprotein E6 and E7 expression, which play an important role in the carcinogenesis of PSCC (37, 50). The viral oncoprotein E7 can bind and inactivate retinoblastoma protein (pRB, a cell cycle regulator), leading to uncontrolled cell cycle progression (42). Disruption of the negative feedback between p16^INK4A^ and pRB due to the inactivation of pRB caused by viral oncoprotein E7 leads to high-level expression of p16^INK4KA^ in PSCC (49, 50). Based on a previous study, p16^INK4A^ was expressed in 79.6% of HPV-positive PSCC compared to 5% in HPV-negative PSCC; thus, p16^INK4A^ is considered a surrogate for HPV infection in PSCC (39). In addition, proteasome-mediated degradation of the tumor suppressor protein p53 by the viral oncoprotein E6 leads to the accumulation of secondary genetic events, including tumor-causing mutations (37, 42). Furthermore, through the activation of telomerase by viral oncoprotein E6 and the combined action of viral oncoprotein E6 and E7, human primitive epithelial cells can achieve immortality (51).

The oncogenic mechanism of HPV-negative PSCC is not yet well understood, and dysregulated p16^INK4A^/cyclin D/RB, p14ARF/MDM2/P53 and PI3K-AKT-mTOR pathways have been reported to be associated with the carcinogenesis of HPV-negative PSCC (42, 52, 53).

### Tumor mutational burden and microsatellite instability for penile cancer

Human somatic mutation-derived expression of cancer rejection antigens is a major driver of the anti-tumor immune response (54). High-level tumor mutational burden and microsatellite instability (MSI) are associated with increased neoantigen expression, adequate tumor-infiltrating lymphocytes (TILs), and upregulated immune checkpoint expression in tumors (55). Therefore, patients with TMB-H and MSI-H are expected to benefit more from ICI therapy, which has been demonstrated in multiple tumors, such as urothelial cancer (24), colorectal cancer (55), non-small cell lung cancer (NSCLC) (56), melanoma (57) and gastric cancer (58). The association between a better response to ICI and TMB-H/MSI-H in carcinomas led to the Food and Drug Administration (FDA) approval of pembrolizumab for advanced unresectable solid tumors of MSI-H or dMMR in 2017, making pembrolizumab the first ICI based on molecular markers rather than clinicopathology (59). Within the context of PSCC, the NCCN guideline recommends pembrolizumab as the second-line treatment for advanced PSCC. However, a study that included 100,000 human tumor genomes revealed a much lower TMB in PSCC than in skin melanoma, urothelial bladder cancer, and NSCLC (60). Similarly, a study of 105 PSCC found that MSI and dMMR were not routine features of penile cancer (61). The rarity of MSI and TMB in metastatic PSCC was also found in a study that included 78 metastatic PSCC (62). Although these findings suggest that ICI may not be a viable therapy in penile cancer, ICI therapy may be effective in patients with a tumor mutational burden greater than 10 mutations per MB, which accounts for 18% of PSCC (52). In addition, the rarity of STK11 mutation and MDM2 proliferation in PSCC (62), which was found to play an important role in resistance to ICI in lung adenocarcinoma (63) and tumor hyperprogression after ICI therapy (64), respectively, also supports the potential application of ICI in penile cancer.

### Tumor-infiltrating lymphocytes in penile cancer

As an important part of the TIME, TILs are involved in the process of anti-tumor immunity and tumor immune escape (65). According to the infiltration status of immune cells into the tumor, the TIME can be divided into three phenotypes: immune desert (no lymphocyte infiltration), immune excluded (stromal infiltration), and immune inflammation (intratumoral infiltration) (66, 67). Within the context of PSCC, compared with intratumor, CD8^+^ T cell and Foxp3^+^ regulatory T cell (T_reg_) numbers are much higher in the stroma of penile cancer, indicating aggregated but not efficiently infiltrating TILs; thus, penile cancer seems to be immune excluded (68). In general, CD8^+^ cytotoxic T cells (CTLs), CD4^+^ helper T cells, and natural killer cells are expected to have anti-tumor activity, while T_reg_ cells, tumor-associated macrophages (TAMs), and myeloid-derived suppressor cells (MDSCs) are associated with tumor immunosuppression (69–74). The roles of various immune cells and their relationship to clinical outcomes have been partially explored in PSCC.

CTLs play a major role in tumor cell killing, in which two steps are needed. First, cancer rejection antigens must be taken up by antigen-presenting cell (APC) and cross-presented to prime naive CD8^+^ T cells, and second, CTLs recognize and kill tumor cells through cancer rejection antigens presented by HLA-I expressed by tumor cells (74, 75). Unfortunately, these two processes can be exploited to achieve immune escape in penile cancer. Within penile cancer, prostaglandin E2 (PGE2), which has been found to contribute to tumor immune escape by inactivating dendritic cells (DC), inhibiting the maturation of DC, and preventing the aggregation of DC to the tumor area in a cyclooxygenase-dependent way (76, 77), was found to be expressed in penile cancer tissue but not in normal penile tissue. In addition, partial loss of HLA-A, an essential component of HLA-I, was also discovered in penile cancer and was associated with worse survival (68). These findings indicate that penile cancer can evade the killing of CTLs through multiple pathways to achieve immune escape. The association between CD8^+^ T cells and clinical outcomes in penile cancer has been explored in a few studies (68, 78, 79). In an observational study that included 213 PSCC patients, it was found that high-level intrastromal CD8^+^ T cells infiltration was associated with reduced lymph node metastasis (LNM) in univariate but not in multivariate regression analysis (68). Another study of 178 PSCC patients found that high-level CD8^+^ T cells infiltration was significantly associated with better disease-specific survival (DSS) (78). Interestingly, compared with HPV-negative penile cancer, the level of CD8+ T cell infiltration was reported to be much higher in HPV-positive penile cancer, which may represent the presence of a stronger anti-tumor response in HPV-positive penile cancer and can partially explain the better prognosis of patients with HPV-positive penile cancer (78, 79).

MDSCs are a group of myeloid-derived suppressor cells with diverse and heterogeneous phenotypes that can suppress innate and adaptive immunity through multiple pathways and are therefore associated with tumor immune escape (80–82). In a study that included 106 newly diagnosed solid tumor patients, it was reported that the level of circulating MDSCs in blood is positively correlated with tumor stage and metastatic tumor burden (83). The association between a high level of MDSCs and poor clinical outcomes in solid tumors was also confirmed in two meta-analyses (84, 85). In the case of penile cancer, researchers of MD Anderson built a genetically modified mouse model of PSCC and found that immunosuppression in murine penile tumors was primarily mediated by MDSCs (86). Although the monoclonal penile tumors in the mouse model cannot represent the heterogeneity within human penile cancer, it directly reveals the important role of MDSCs in tumor immune escape in penile cancer. A recently developed xenograft model that can be humanized can overcome this deficiency and may help us gain better insight into the immune microenvironment of penile cancer in the future (87).

CD4^+^CD8^+^Foxp3^+^ Treg cells are recognized as immunosuppressive cells. Different from the high basal level expression of CD28 and low basal level expression of cytolytic T lymphocyte-associated antigen 4 (CTLA-4) in traditional T cells, T_reg_ cells constitutively express high-level CTLA-4, which is essential for the immunosuppressive function of T_reg_ cells (88, 89). Within penile cancer, several studies have found conflicting associations between T_reg_ cells (CTLA-4) and clinical outcomes. In a study of 122 patients diagnosed with usual-type PSCC, the presence of peritumoral infiltration of T_reg_ cells was found to be significantly associated with unfavorable clinical outcomes (90). In contrast, another study revealed a correlation between high-density CTLA-4 expression in the tumor stroma and better DSS (78). Conflicting results suggest that it is limited to correlate T_reg_ cell counts with clinical outcomes alone, as a key factor in tumor prognosis is the ratio of effective T cells to T_reg_ cells infiltrating the tumor (91). Compared with HPV-negative PSCC, higher levels of CTLs and T_reg_ cell infiltration coexisted in HPV-positive PSCC (78, 79), demonstrating that stronger tumor killing and immunosuppression can coexist in the TIME of PSCC.

TAMs are highly plastic and can generally be divided into M1 macrophages with anti-tumor functions and M2 macrophages with tumor progression-promoting functions (92). In vulvar SCC (93) and head and neck SCC (HNSCC) (94), a high density of intratumoral CD68^+^ TAMs was found to correlate with tumor progression and poor prognosis. In contrast, in PSCC, a high level of CD68^+^ TAMs was found to be associated with improved CSS (78). A high level of CD163^+^ TAMs (M2) was found to be associated with worse prognosis and higher tumor stage in NSCLC (95), whereas in penile cancer, a high level of CD163^+^ TAMs was associated with increased LNM rather than poor survival (68). The high plasticity of TAMs and the limitation of using CD markers alone to differentiate TAMs may contribute to the contradictory phenomenon shown in penile cancer with traditionally thought of the role of TAMs (36).

### PD1 axis in penile cancer

After T cells activation, the expression of PD-1 on T cells is upregulated and mainly binds to its ligand (PD-L1) on the surface of APC, which leads to T cells dysfunction, also known as T cells exhaustion, thereby moderating T cells activation to achieve immunosuppression (65). The interrelationship between PD1 and PDL1, known as the PD1 axis, plays a vital role in maintaining immune balance. Unfortunately, this physiological immunosuppressive process can be exploited by cancer cells to achieve tumor immune escape by upregulating the expression of PDL1 on the tumor cell surface (96). In penile cancer, PDL1 has been reported to be expressed in 40-69% of primary PSCCs. The highest frequency of expression of PDL1 (69%) was reported by a study that included 40 PSCC (97), while another study including 53 PSCC reported the lowest frequency (40%) (98). In addition, in a study including 37 PSCC, the concordance of PDL1 expression between primary and metastatic penile tumor tissues was revealed by Spearman’s rank correlation coefficient (ρ = 0.72, 0.032 < P < 0.036), which can help us speculate on the high expression of PDL1 in metastases. Although two studies with small sample sizes of 40 and 53 PSCC, respectively, found no statistically significant association between PDL1 expression and survival outcome (97, 99), the increased HR and wide 95% CI (HR, 2.13; 95% CI, 0.67-7.71; P = 0.199) in one study suggest that the statistical insignificance may be due to the low test power of the small sample size (97). In most studies, patients with diffusely expressed PDL1 penile carcinoma tissue had higher LNM and worse survival prognosis than those with PDL1-negative or border-positive (68, 98, 100). In addition, PDL1 is highly expressed in usual-type PSCC, whereas PDL1 is negatively expressed in warty or verrucous PSCC with better survival outcome, which also supports the negative correlation between PDL1 expression level and survival outcome in penile cancer (98).

In conclusion, penile cancer has a high rate of PDL1 expression that is correlated with poor clinical outcomes, suggesting the potential benefit of ICI in the treatment of penile cancer. However, due to the rarity of PSCC, our understanding of the TIME of PSCC is limited and it is difficult to precisely predict who is more likely to benefit from ICI. Large studies are needed to better elucidate the TIME to help us explore immunotherapy for penile cancer patients in the future.

## Immune-based treatments for penile cancer

**Figure 2:**
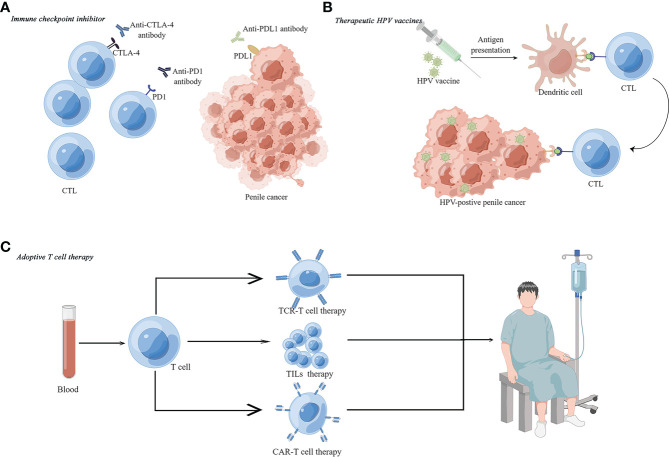
Immune-based therapies for penile cancer. **(A)** Immune checkpoint inhibitors (ICIs) include anti-PDL1, anti-PD1 and anti-CTLA4 agents. **(B)** Therapeutic HPV vaccine can activate the immune system to kill HPV-positive penile cancer. **(C)** Adoptive T-cell therapy (ATC) for penile cancer, such as tumor-infiltrating lymphocytes (TILs) therapy, chimeric antigen receptor therapy (CAR-T) and T cell receptor (TCR) therapy.

Although rarely used in penile cancer, immune-based treatments including ICI, therapeutic HPV vaccines and ATC are potentially effective treatments for penile cancer based on current understanding of penile cancer (**Figure 2**).

### Immune checkpoint inhibitor for penile cancer

In 2011 and 2014, ipilimumab and pembrolizumab/nivolumab were approved by the FDA for advanced unresectable melanoma, becoming the first CTLA-4 inhibitor and PD-1 inhibitor, respectively (101, 102). Then, in 2016, atezolizumab was approved for second-line systemic treatment of metastatic urothelial carcinoma, becoming the first approved PDL1 inhibitor by the FDA (24). ICIs have been approved for various genitourinary tumors and squamous cell carcinomas (23–30, 102). In penile cancer, pembrolizumab is recommended for second-line treatment of recurrent or/and metastatic advanced penile cancer (13). The data on the effect of ICI in penile cancer, however, are mainly from case reports and basket trials due to the rarity of PSCC (31–33). In a case report, 2 patients with metastatic PSCC who progressed after chemoradiotherapy responded to pembrolizumab and achieved long-term clinical benefit (31). In another case report, a case of chemoradiotherapy-refractory advanced PSCC responded to nivolumab (13). Penile cancer is quite uncommon; hence, clinical trials investigating the effect of ICIs are mainly basket trials. In a phase 2 basket trial for rare urogenital malignancies (NCT03333616) that included 5 PSCC, no patient responded to the combination of pembrolizumab and nivolumab, while 2 patients had stable disease and 3 patients had progressive disease (33). In a case series from a phase II basket trial (NCT02721732) that included 3 patients with advanced PSCC, 1 patient with MSI-H had a partial response (PR) to pembrolizumab (32).

According to the findings of a high PDL1 expression rate in PSCC and a negative correlation between PDL1 expression level and survival in PSCC. It is rational to apply ICI to PSCC. Several clinical trials are being conducted to investigate the effects of ICI in various stages of PSCC. For example, avelumab (anti-PDL1) for PSCC patients with progressive disease after platinum-based chemotherapy is ongoing in a phase 2 trial that included 24 patients (NCT03391479) (103), and avelumab is being tested as maintenance therapy following chemotherapy for PSCC patients with surgically unresectable disease in another phase 2 trial (NCT03774901) (104). In addition, given the synergistic effect of radiotherapy/chemotherapy and immunotherapy shown in tumor treatment (105), clinical trials of the combination of ICI and radiotherapy/chemotherapy for PSCC are also underway, such as atezolizumab plus radiotherapy for surgically unresectable PSCC (NCT03686332) (106) and pembrolizumab plus cisplatin (or carboplatin) and 5-FU as the first line of advanced PSCC (NCT04224740) (107). Ongoing trials of ICI in PSCC (not including basket trials) are summarized in **Table 1**.

**Table 1:** Ongoing clinical trials of ICI in PSCC (basket trials not included).

Identifier	Phase	Arm(s)	Estimated participants	ICI	Combination	Disease status	Primary endpoint	Ref
NCT03391479	Phase 2	Single arm	24	Avelumab (anti-PDL1)	NA	Locally advanced or metastatic PSCC who are unfit for or progressed on platinum-based chemotherapy	ORR	(103)
NCT03774901	Phase 2	Single arm	32	Avelumab (anti-PDL1)	NA	Unresectable locally advanced or metastatic PSCC who responded to first line platinum-based chemotherapy	PFS	(104)
NCT03686332	Phase 2	2 arms	32	Atezolizumab (anti-PDL1)	Radiotherapy	Unresectable advanced PSCC (T4 or N2/N3 or M1)	PFS	(106)
NCT04224740	Phase 2	Single arm	33	Pembrolizumb (anti-PD1)	Ciplastin plus 5-Fluouracil	First-line Systemic Therapy in Advanced PSCC (T4 or N3 or M1)	ORR	(107)
NCT04231981	Phase 2	Single arm	18	INCMGA0012 (anti-PD1)	NA	Locally advanced unresectable or metastatic PSCC (T4 or N3 or M1)	ORR	(108)

ICI, immune checkpoint inhibitor; ORR, objective response rate; PFS, progression free survival.

In conclusion, there are few reports on the effect of ICI treatment on PSCC. ICI is currently mainly used in patients who have failed surgery, radiotherapy and/or chemotherapy, and some clinical trials of ICI for PSCC are underway. However, most ongoing clinical trials are single-arm trials with small samples, which may limit their validity. Large, controlled clinical trials comparing ICI with current standard care of PSCC are needed.

### HPV vaccine

Due to the high prevalence of HPV infection in PSCC and the role of HPV in tumorigenesis, the HPV vaccine naturally has a potential role in the prevention and treatment of PSCC. In women, preventive HPV vaccines were proven to reduce the risk of cervical cancer (an HPV-related disease) by 87% in a large observational study in UK (109). The role of preventive HPV vaccination in reducing the incidence of PSCC is unclear, but the proportion of men vaccinated against HPV is very low, approximately 4% in 2019 (110). Unlike prophylactic HPV vaccines to protect the population from HPV infection, therapeutic HPV vaccines are designed to clear tumors through the immune system. Due to the long-term HPV activity in HPV-infected cells, HPV-positive PSCC continues to express viral oncoproteins E6 and E7, which can be targets for anti-tumor therapy (37, 50). By driving the immunogenicity of viral oncoproteins E6 and/or E7 in HPV-positive PSCC, therapeutic HPV vaccine can promote CTL-mediated HPV-infected or HPV-transformed cell killing, which has been proven to be effective in multiple HPV-driven neoplasms and malignancies (111). The Lm-LLO-E7 vaccine, which can secrete the HPV-16 E7 antigen fused to a nonhemolytic fragment of the Lm protein listeriolysin O, led to 7/15 patients with stable disease and 1/15 patients with partial response in a study that included 15 patients with previously treated metastatic, refractory or recurrent cervical cancer (112). In a phase 3 pivotal trial, VGX-3100, a synthetic plasmid targeting HPV-16 and HPV-18 E6 and E7 oncoproteins, also demonstrated good efficacy and safety in histopathological regression in women with high-grade precancerous cervical dysplasia (113). These studies demonstrated the efficacy of therapeutic HPV vaccines in the treatment of HPV-related cancer; however, there are currently no published findings on therapeutic HPV vaccines for PSCC.

### Adoptive T cell therapy

Adoptive T cell therapy, which includes antigen receptor-engineered T cell therapy (TCR-T and CAR-T) and TILs transfer therapy, is an emerging tumor treatment modality and has become an important part of tumor immunotherapy. TCR-engineered T cells targeting E7 have been reported to mediate the regression of human papillomavirus cancers in a mouse model (114). The efficacy of TCR-T therapy in human PSCC was also confirmed. A total of 6/12 (50%) patients had objective responses, and 5/12 (41.7%) patients had stable disease after TCR-T cell therapy targeting the viral oncoprotein E7 in a phase 1 trial that included 12 metastatic HPV-associated epithelial cancers (11 patients with PSCC) (115). Unlike TCR-T cell therapy, which kills tumor cells depending on the tumor-rejecting antigens presented by HLA-I, chimeric antigen receptor T therapy (CAR-T) acts directly through tumor-rejecting antigens expressed on the surface of tumor cells to achieve killing of tumor cells, thus avoiding the immune escape of tumor cells by downregulating HLA-I expression, which was found in PSCC. However, no research on the application of CAR-T cell therapy to PSCC has been reported thus far. TILs transfer therapy for PSCC was shown to be feasible *in vitro*; in 11 out of 12 patients with PSCC, TILs were expanded by high concentrations of IL-2 from resected positive metastatic lymph nodes, and 5 out of 11 expanded TILs samples had anti-autologous tumor activity (116). However, there are currently no reports of TILs transfer therapy *in vivo*.

## Conclusion

Currently, advanced PSCC has limited treatment options and poor clinical outcomes. Due to the rarity of the disease, the TIME of penile cancer is currently not well described, which limits our ability to accurately identify optimal immune-based treatments. However, the high expression of PD-L1 in penile cancer tissues supports the potential application of ICIs in penile cancer, and several clinical trials are underway. In addition, due to the unique role of HPV infection in HPV-positive penile cancer patients, therapeutic HPV vaccine is also a potential treatment modality. In addition, ATC therapy also showed efficacy in treating advanced penile cancer in some early clinical trials.

## Author contributions

Writing and figures, YT, XH, and KW. Concept and proof reading, XL. All authors contributed to the article and approved the submitted version.

## Funding

This work was supported by the Sichuan Science and Technology Program (2022YFS0133).

## Acknowledgments

We thank all who provided advice and assistance with the concept and writing of this review.

## Conflict of interest

The authors declare that the research was conducted in the absence of any commercial or financial relationships that could be construed as a potential conflict of interest.

## Publisher’s note

All claims expressed in this article are solely those of the authors and do not necessarily represent those of their affiliated organizations, or those of the publisher, the editors and the reviewers. Any product that may be evaluated in this article, or claim that may be made by its manufacturer, is not guaranteed or endorsed by the publisher.
